# *Trichoderma virens* Gl006 and *Bacillus velezensis* Bs006: a compatible interaction controlling Fusarium wilt of cape gooseberry

**DOI:** 10.1038/s41598-020-63689-y

**Published:** 2020-04-22

**Authors:** L. F. Izquierdo-García, A. González-Almario, A. M. Cotes, C. A. Moreno-Velandia

**Affiliations:** 1 Corporación Colombiana de Investigación Agropecuaria – AGROSAVIA, Centro de Investigación Tibaitatá, Km 14 vía Mosquera - Bogotá, Mosquera, Colombia; 2 Facultad de Ciencias Agrarias, Universidad Nacional de Colombia, Bogotá, Colombia

**Keywords:** Applied microbiology, Microbe

## Abstract

The combination of *Trichoderma virens* Gl006 and *B. velezensis* Bs006 as a consortium has high potential to control Fusarium wilt (FW) of cape gooseberry (*Physalis peruviana*) caused by *Fusarium oxysporum* f. sp. *physali* (Foph). However, the interactions between these two microorganisms that influence the biocontrol activity as a consortium have not been studied. Here, we studied the interactions between Gl006 and Bs006 that keep their compatibility under *in vitro* and greenhouse conditions. Antagonism tests between Gl006 and Bs006 inoculated both individually and in consortium against Foph strain Map5 was carried out on several solid media. The effect of supernatant of each selected microorganism on growth, conidia germination, biofilm formation and antagonistic activity on its partner was also studied. Biocontrol activity by different combinations of cells and supernatants from both microorganisms against Fusarium wilt was evaluated under greenhouse conditions. *In vitro* antagonism of the consortium against Foph showed a differential response among culture media and showed compatibility among BCA under nutritional conditions close to those of the rhizosphere. The supernatant of Bs006 did not affect the antagonistic activity of Gl006 and vice versa. However, the supernatant of Bs006 promoted the biocontrol activity of Gl006 in a synergistic way under greenhouse, reducing the disease severity by 71%. These results prove the compatibility between *T. virens* Gl006 and *B. velezensis* Bs006 as a potential tool to control Fusarium wilt of cape gooseberry.

## Introduction

*Fusarium oxysporum* is the causal agent of Fusarium wilt disease in several species of cultivated plants worldwide. This phytopathogen is in the fifth place within the top ten of the most important plant pathogens due to its effects in crops of economic importance causing severe losses^1^. Fusarium wilt is the main limitation of cape gooseberry (*Physalis peruviana*) crop in Colombia^2^. Wilt symptoms in field include wilting of top leaves, stunting of plants, lateral yellowing of branches and leaves, some plants present both dry and alive branches. Plants finally become yellow and die, resulting in plant losses and reduced fruit yield. This phytosanitary problem has also caused the migration of cropped areas within the country, but the problem persists in old and new cropped areas^2^. Currently, there are no registered phytosanitary products for the control of this disease in cape gooseberry.

In this context, biological control has emerged as an environmentally sustainable alternative. However, classical biological control in which just one biological control agent (BCA) is used has shown high variability among tests. Considering that biocontrol agents are living organisms and they may not be active in all agroecosystems^3^, many of the recent studies have focused on using combinations of BCA to increase the efficacy against phytopathogens. Those combinations are known as consortia. A consortium is a microbial association of two or more microorganisms, which could be archaea, fungi, bacteria, virus or algae^4,5^. However, there can be either direct or indirect interactions among the different populations of BCA in consortium, which could result in negative or positive effects on the biocontrol efficacy^6^. Therefore, in a combination of two BCA, the biocontrol activity can be increased, reduced or similar compared to individual activity.

Several studies have evaluated the efficacy of microorganisms in consortium, but few have evaluated how microorganisms interact each other and nor have identified the mechanisms by which the consortium has higher biocontrol activity than the individual components. Knowing these interactions will allow to design more effective consortia and take advantage of its potential. In the earliest studies of microbial consortia, the selection of pairs or mixtures was done according to the individual potential but did not mention whether there was an antagonistic or suppressive effect on its partner. The recommendation for current studies is to evaluate how the components of a consortium interact with each other, including how they interact at functional levels and to determine the predominant component of the consortium^7^.

There are studies about the synergistic or additive effects of application of microbial consortia containing two, three and even four microorganisms, the most frequent in these studies are *Trichoderma* spp., *Pseudomonas* spp., *Bacillus* spp., and *Gliocladium* spp. Mixtures of fungus - fungus, fungus - bacteria and bacteria – bacteria, combination of microorganisms belonging to the same genus or strains of the same species have been evaluated^7^. Some examples of these studies are the reduction of diseases caused by *Fusarium solani* and *Rhizoctonia solani* using *Bacillus subtilis* MBI600 and *Rhizobium tropici* UMR 1899 in bean^8^; the use of *Trichoderma harzianum* DB11 and *Gliocladium catenulatum* to control *Phytophthora cactorum* in strawberry^9^; the decrease of the mortality of pea plants due to *Sclerotinia sclerotiorum* using a consortium of *Pseudomonas* sp., *Bacillus* sp. and *Trichoderma* sp^10^; the combination of *B. subtilis* and *Pseudomonas fluorescens* against *F. solani* in bell pepper^11^; the reduction of the incidence of the disease caused by *Rhizoctonia bataticola* in cotton using a consortium of *Azospirillum* sp. AZ204 and *P. fluorescens* Pf1^12^; and the combination of *T. harzianum* Tr16 and *Pseudomonas* sp. Ps14 against *F. oxysporum* in cucumber^13^.

*Trichoderma* spp. and *Bacillus* spp. are among the most commonly used microorganisms in biological control against *F. oxysporum*^14^ but few studies have evaluated consortia based on *Trichoderma* spp. and *Bacillus* spp. There have been evaluated different isolates of *Trichoderma* spp. and *Bacillus* spp. against *R. solani* and as plant growth promoters on cucumber and beans^15^. However, none of the combinations improved the efficacy showed by the individual activity of three isolates of *Trichoderma* spp. and only plant growth promotion by the consortium was observed. Some studies about the potential combination of *Trichoderma* spp. and *Bacillus* spp. have been conducted mainly *in vitro*. For instance, Wu *et al*.^16^ evaluated the antimicrobial effect of co-culturing *Trichoderma asperellum* GDFS1009 and *Bacillus amyloliquefaciens* ACCC11060 in BP broth (beef extract 0.3% and peptone 0.5%) finding the greatest effects against *Botrytis cinerea* by the supernatant from the co-culture than individual treatments. Other study demonstrated that *in vitro* co-culture of *T. asperellum* GDFS1009 and *B. amyloliquefaciens* 1841 induced the production of compounds not detected under pure cultures, attributed to the competition between them^17^. According to these findings, studies between *Trichoderma* spp. and *Bacillus* spp. are related to production of antimicrobial compounds in co-culture but the *in vivo* interactions between cells-supernatants or cells-cells of biocontrol agents have not been addressed. To the best of our knowledge, the work of Silva^18^ is the only one reporting the evaluation of *in vitro* compatibility between strains of *Trichoderma* spp. and strains of *B. subtilis* and *Bacillus cereus* as a method to screening potential consortia against FW in cape gooseberry. However, there are no published works on *in vivo* evaluations.

In previous studies the rhizobacteria *Bacillus velezensis* Bs006 was selected for its potential to control Fusarium wilt, but it has shown variable efficacy among tests^2^. Also, the combination of *Trichoderma virens* Gl006 and *B. velezensis* Bs006 increased the biocontrol activity and maintained consistently the effectiveness, against Fusarium wilt of cape gooseberry (Izquierdo-García *et al*. Unpublished). However, the interactions between these BCA remain unknown which would help to establish guidelines to make the consortium more effective. In this context, the aim of this study was to evaluate the compatibility among the biocontrol agents Gl006 and Bs006 and to know their interactions.

## Results

### Interactions between biological control agents under *in vitro* conditions

#### Effect of supernatants on the growth of biological control agents

**Figure 1:**
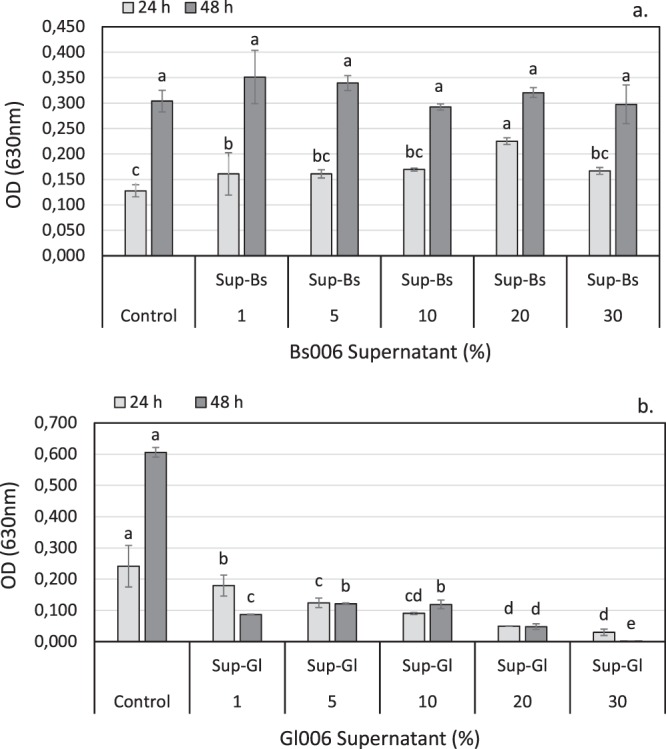
(**a**) Growth of *T. virens* Gl006 in PDB medium supplemented with different concentrations (1, 5, 10, 20 and 30%) of *B. velezensis* Bs006 cell-free supernatant. (**b**) Growth of *B. velezensis* Bs006 in LB medium supplemented with 1, 5, 10, 20 and 30% of *T. virens* Gl006 cell-free supernatant. Growth was expressed as optical density (OD_630 nm_) measured in a microplate reader at 24 and 48 hours. Columns with the same letter are not significantly different according to Tukey test (α = 0.05) at each time of evaluation. Bars on the columns represent standard deviation (n = 3).

Soaking the conidia of *T. virens* Gl006 during 48 h in Bs006-sterile supernatant solution at different concentrations (1 to 30%) did not affect its growth (Supplementary material - Figure S1a). It was observed that the concentrations of 1 and 20% stimulated the growth of Gl006 at 24 hours of exposition, but this effect was transient since at 48 hours there were no significant differences among the treatments (Fig. 1a). On the other hand, all concentrations of GI006 supernatant delayed Bs006 growth in a concentration dependent manner, starting at eight hours of fermentation (Fig. 1b, Supplementary material - Figure S1b).

#### Germination of Gl006 conidia and viability of Bs006 treated with supernatants

Germination of the exposed conidia of Gl006 to Bs006-supernatant showed values close to 90% on water-agar medium after 24 hours of incubation. Thus, none of the Bs006-supernatant concentrations caused negative effects on Gl006 conidia after six hours of exposure. Same results were observed regarding the viability of Bs006 cells exposed to the Gl006-supernatant and none of the concentrations affected to Bs006 (data not shown).

#### Effect of supernatants on the antagonistic activity

**Figure 2:**
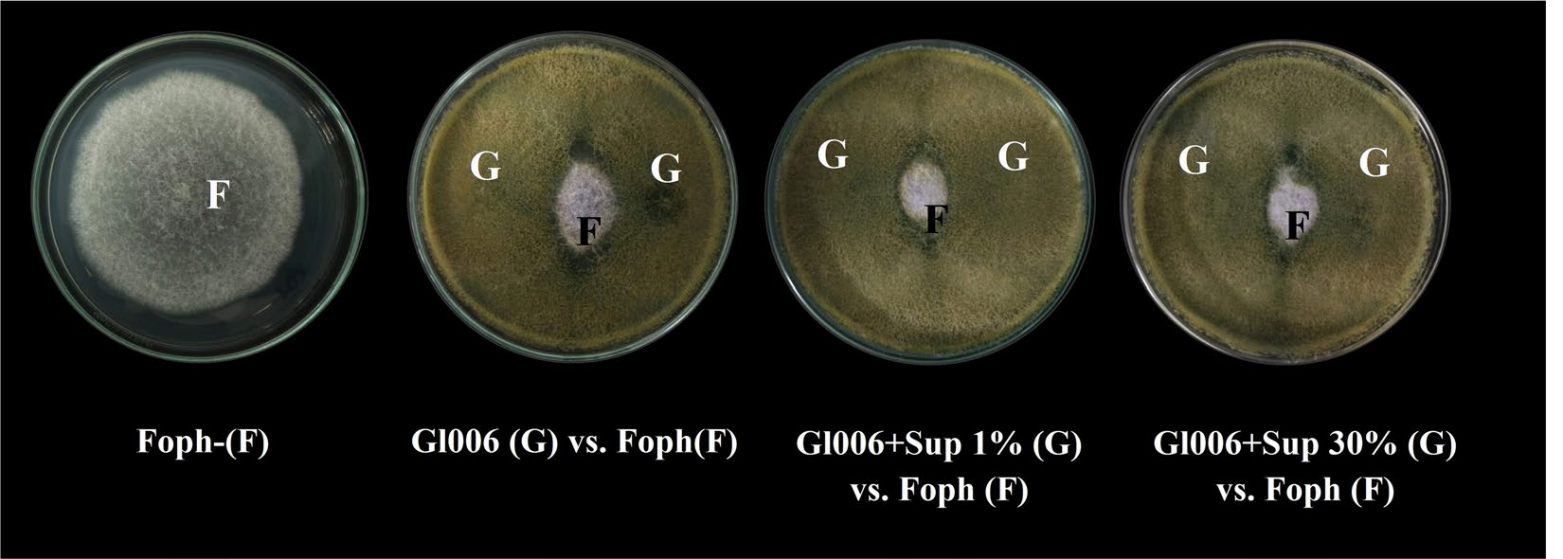
Antagonistic activity of *T. virens* Gl006 after being exposed to Bs006-supernatant in dual confrontation test against *F. oxysporum* f. sp. *physali* strain Map5 (Foph). Gl006 (G) conidia were exposed to Bs006 cell-free supernatant (Sup) from 1 to 30%. Growth inhibition of Foph was measured according to diametral growth in the positive control - F in PDA medium, after seven days of incubation at 25 °C.

**Figure 3:**
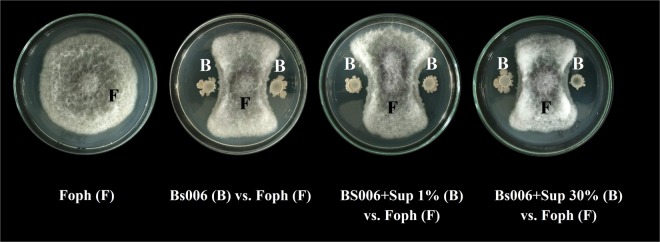
Antagonistic activity of Bs006 after being exposed to Gl006-supernatant in dual confrontation test against *F. oxysporum* f. sp. *physali* strain Map5 (Foph). Bs006 (B) cells exposed for six hours to Gl006 cell-free supernatant (Sup) from 1 to 30%. Growth inhibition of Foph was measured according to diametral growth in the positive control - F in PDA medium after seven days of incubation at 25 °C.

Antagonistic activity of Gl006 was not affected after six hours of exposure of conidia to Bs006-supernatant in concentrations of 1 to 30%. Inhibition of Foph growth by Gl006-treated conidia was in a range of 76 to 78% (Fig. 2). Similar results were observed when Bs006 cells were exposed to the Gl006-supernatant, since exposed cells reduced the growth of Foph in a range of 52 to 54% (Fig. 3).

#### Biofilm formation by B. velezensis Bs006 treated with Gl006-supernatant and under co-culture

**Figure 4:**
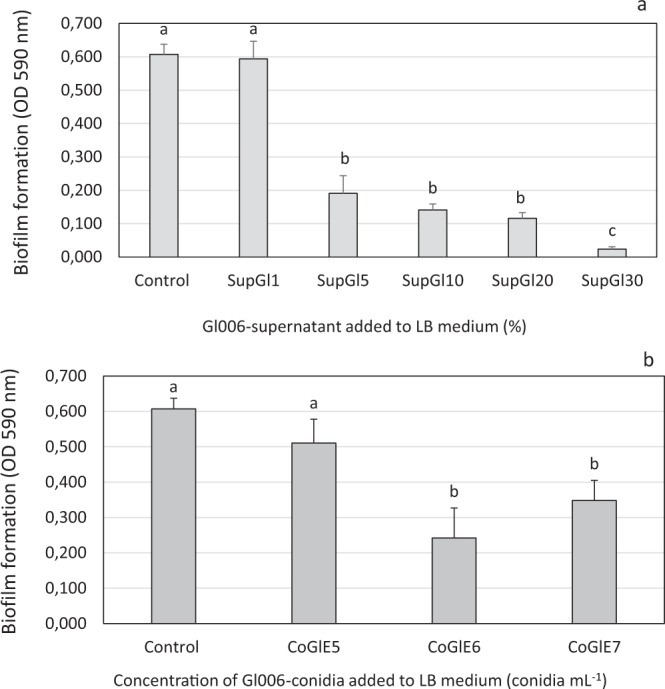
Biofilm formation of *B. velezensis* Bs006 in presence of *T. virens* Gl006 supernatant (**a**) and conidia (**b**). *B. velezensis* Bs006 was grown for 24 h in LB medium supplemented with Gl006-supernatant from 1 to 30% (SupGl1 to SupGl30) or viable conidia of *T. virens* Gl006 at 10^5^ to 10^7^ conidia.mL^−1^ (CoGlE5 – CoGlE7). Control: Growth of Bs006 in LB free of Gl006-supernatant and conidia. Columns with the same letter are not significantly different according to Tukey test (α = 0.05). Bars on the columns represent standard deviation (n = 9).

It was observed that continuous exposition of Bs006 to Gl006-supernatant for 24 hours affected the ability of the bacteria to form biofilms in a concentration dependent manner. The exposure of Bs006 to 1% of Gl006-supernatant did not show negative effects on biofilm formation, but higher concentrations of the supernatant affected significantly this trait (Fig. 4a). Similar results were observed regarding the evaluation of co-cultures of Gl006 with Bs006, where the lowest concentration of Gl006 (1 × 10^5^ conidia.mL^−1^) did not affect biofilm formation by Bs006, but this performance decreased when the bacteria grew with Gl006 in the same medium at 1 × 10^6^ and 1 × 10^7^ conidia.mL^−1^ (Fig. 4b).

#### Effect of co-cultures of Bs006 and Gl006 on the antagonistic activity on different solid media

**Figure 5:**
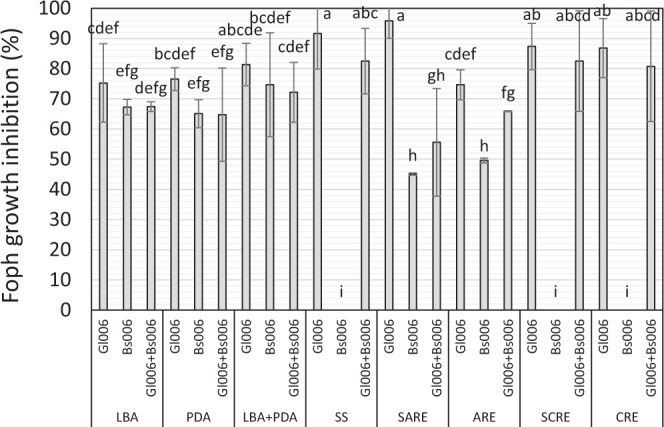
Growth inhibition of Foph colony in solid media by Gl006, Bs006 and the consortium. (ARE), artificial root exudates. soil solution + synthetic root exudates (SARE), soil solution (SS). Luria Bertani-Agar (LBA), Potato-Dextrose-Agar (PDA), PDA (50%) + LBA (50%) (LBA+PDA), Cape gooseberry root exudates (CRE), soil solution + Cape gooseberry root exudates (SCRE). Bars on the columns represent standard deviation between experimental units of three replicates in the time (n = 12). Columns with the same letter are not significantly different according to Tukey test (α = 0.05).

The highest percentage of growth inhibition of *F. oxysporum* Foph on solid media was caused by Gl006 alone (>70%) in all culture media. Differential effect among culture media was observed in dual confrontation tests of Gl006 and Bs006 co-culture against Foph. Thereby, the diameter of the Foph colony was similarly reduced by the treatments Gl006, Bs006 and the combination of Gl006 with Bs006 on synthetic media (LB, PDA and the combination of LB with PDA) (Fig. 5), suggesting that under these conditions there was a neutral interaction between both biocontrol agents. In contrast, Bs006 did not grow and did not reduce Foph growth on soil solution-agar (SS), cape gooseberry root exudates prepared on SS-agar (SCRE) and cape gooseberry root exudates-agar (CRE) media, whereas Gl006 colonized the surface of all these media and inhibited *Fusarium* in both treatments, Gl006 individual and in consortium with Bs006 (Fig. 5), suggesting that Foph inhibition in such conditions was caused by Gl006. But on the other side, it was observed that these three treatments reduced the growth of Foph in media containing only artificial root exudates (ARE) or artificial root exudates prepared in soil solution (SARE), with Gl006 showing the highest growth inhibition of Foph (96 and 75% respectively) (Fig. 5). Bs006 grew and inhibited the growth of *Fusarium* between 45 to 50% on ARE and SARE solid media. In this bioassay there was no additive or synergistic effect by the consortium.

On the other hand, the co-inoculation of Foph, Gl006 and Bs006 on solid media (i.e. all together in the same suspension) showed up to 100% inhibition of Foph, also depending on the culture medium. Thereby Bs006 grew only on synthetic media, while growth of the two biocontrol agents was observed on the media containing soil solution and root exudates (ARE and SARE) (Table 1). Moreover, it was found that Bs006 inhibited the growth of Gl006 by 60% on synthetic media PDB and LB in the dual confrontation test of both BCA. Whilst the inhibition was 50% on SARE and ARE media. In contrast, Bs006 did not inhibit the growth of Gl006 on SS and cape gooseberry root exudates media CRE and SCRE (Supplementary material - Figure S2).

**Table 1:** Growth of microorganisms inoculated in mixture on different culture media.

Culture medium	*T. virens* Gl006	*B. velezensis* Bs006	Foph
ARE	+	+	−
CRE	+	−	−
SS	+	−	−
SARE	+	+	−
SCRE	+	−	−
LBA	−	+	−
PDA	−	+	−
LBA+PDA	−	+	−

A drop of 10 µL of suspension with the mixture of Gl006 (1 × 10^6^ conidia.mL^−1^), Bs006 (1 × 10^8^ cells.mL^−1^) and Foph (1 × 10^5^ microconidia.mL^−1^) was inoculated in the center of the Petri dish (90 mm). The evaluation of the growth was made after seven days of incubation at 25 °C. LBA - Luria Bertani Agar, PDA – Potato Dextrose Agar, LBA+PDA - 50% each, Artificial Root Exudates - ARE^28^, Root Exudates from cape gooseberry - CRE, Soil Solution - SS, ARE and CRE media prepared on SS - SARE and SCRE, respectively.

### Microscopic observations of the Gl006 and Bs006 interactions

#### Interactions on solid media

**Figure 6:**
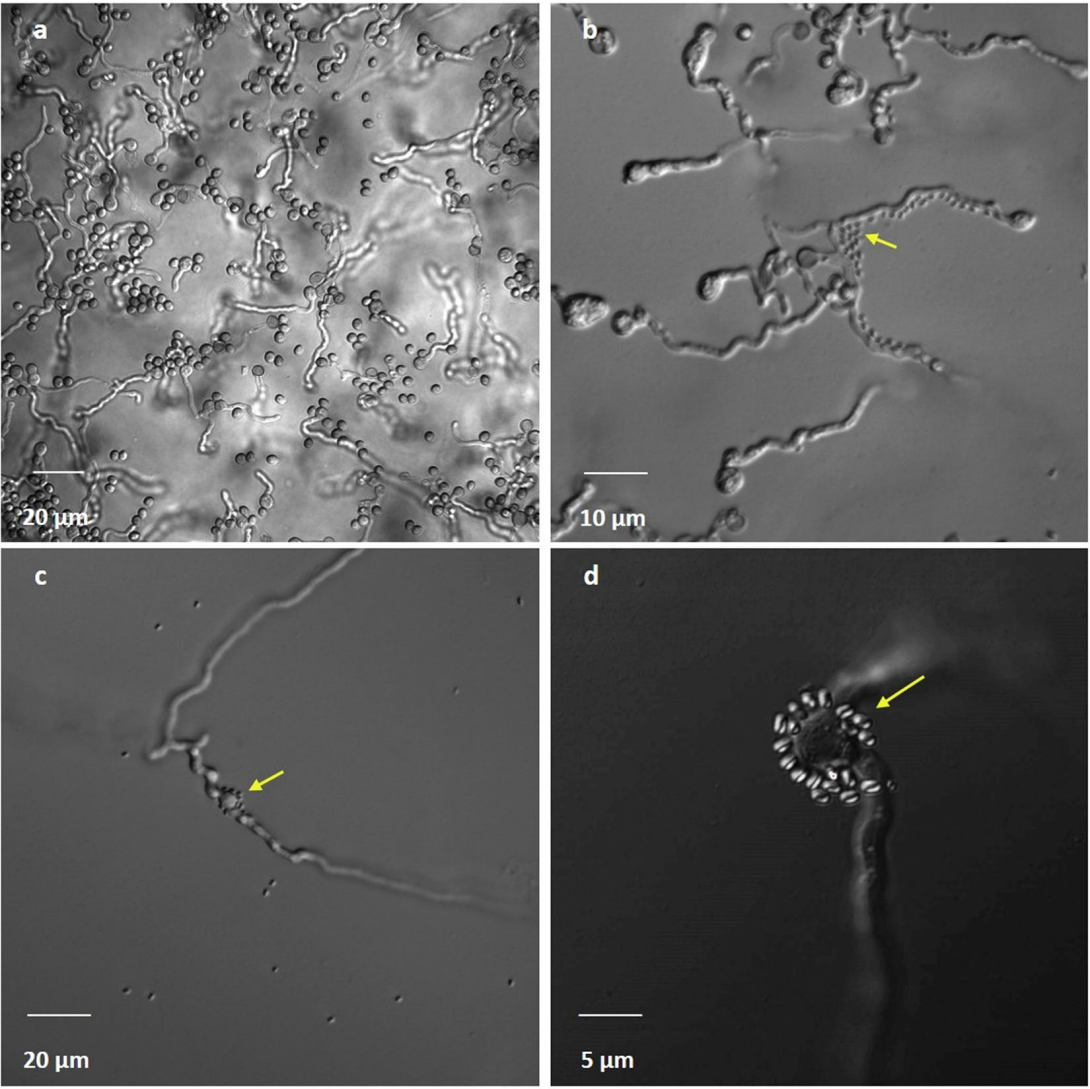
Microscopy observations of the interaction between *T. virens* Gl006 and *B. velezensis* Bs006 in jellified cape gooseberry root exudates under fluorescent microscope. (**a**) Germinated conidia of *T. virens* Gl006 in absence of *B. velezensis*. (**b–d**) Interaction between Gl006 conidia and Bs006 cells. Yellow arrows indicate Bs006 spores at the beginning of biofilm formation on Gl006 surface after 72 hours contact.

Inoculation of Gl006 and Bs006 on jellified cape gooseberry root exudates allowed to observe that Gl006 conidia germinated and their hyphae extended to the interaction zone of both microorganisms (Supplementary material - Figure S3). Bs006 cells were adhered both to the non-germinated and germinated conidia. Bs006 cells also were adhered to the Gl006 hyphae forming aggregates like a biofilm (Fig. 6). No damages on fungal structures were observed.

#### In planta interactions

**Figure 7:**
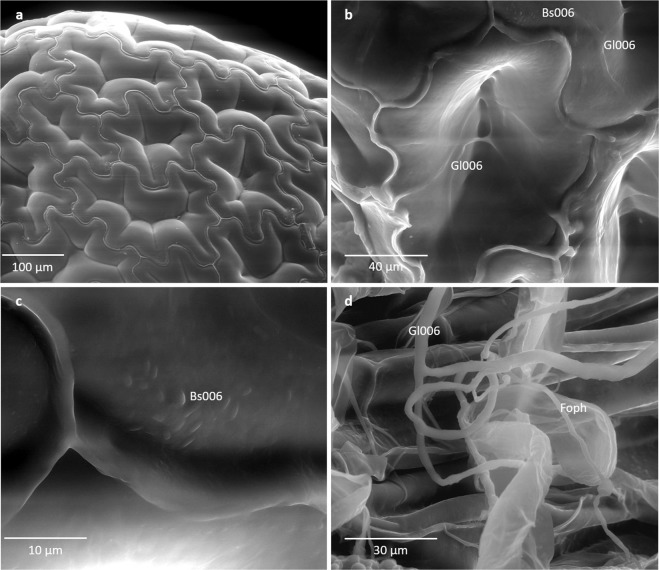
Environmental scanning microscopy images of non-germinated and germinated seeds of cape gooseberry inoculated with *T. virens* Gl006 and *B. velezensis* Bs006. (**a**) Surface of seed coat without inoculations, (**b** and **c**) co-inoculated seed with Gl006 and Bs006, (**d**) co-inoculated seedling with Bs006, Gl006 and Foph. **Bs006:** *B. velezensis* Bs006 cells, **Gl006:** *T. virens* Gl006 hypha, **Foph*:**** F. oxysporum* f. sp. *physali*-Map5. Samples were observed at 24 hours after inoculation and incubation at 25 °C.

It was observed that Gl006 colonized the seed-surface forming a net (Fig. 7). Mycelium of Gl006 was observed around the root hairs. However, some conidia did not germinate at 24 h after inoculation. On the other hand, Bs006 made a biofilm on the surface of both the seed and the root (Fig. 7). When Gl006 and Bs006 were inoculated together on the host surface, it was noticed that both microorganisms colonized the surface of the plant tissue, Gl006 formed a net and Bs006 a biofilm (Fig. 7).

### Biocontrol activity of Gl006 and Bs006 consortium against Fusarium wilt disease under greenhouse conditions

**Figure 8:**
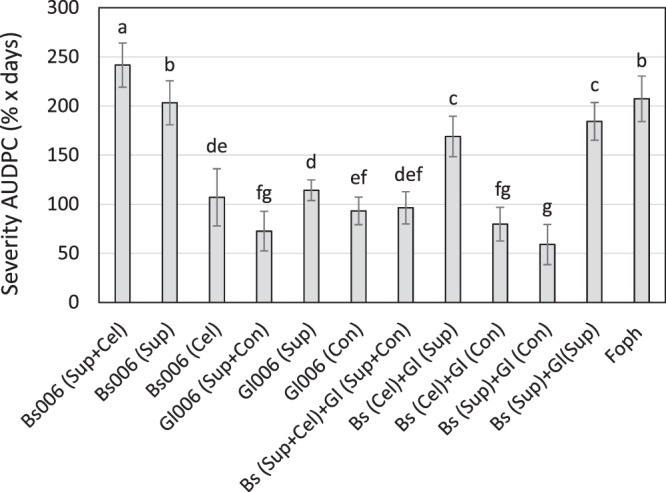
Effect of the combination of cells and supernatants of Gl006 and Bs006 on the progress of Fusarium wilt severity. Columns with the same letter are not significantly different according to Duncan's multiple range test (α = 0.05). Con = Conidia, Cel = cells, Bs = *B. velezensis* Bs006, Gl = *T. virens* Gl006. Sup = cell-free supernatant.

Application of Bs006 cells suspension prepared in Gl006-supernatant solution and vice versa and the combination of cells and supernatants of both microorganisms to the soil, showed that Bs006 and Gl006 cells, supernatants, and Gl006 conidia resuspended in Bs006-supernatant significantly reduced the progress of both Fusarium wilt incidence (Supplementary material - Figure S4) and severity (Fig. 8), as compared to the negative control. Interestingly, Bs006 cells suspended in its own supernatant showed reduced effectivity against FW, compared to the application of Bs006-supernatant alone and the Bs006 cells suspension in water (Fig. 8, Table 2). Whereas the suspension of Bs006 in Gl006-supernatant decreased the efficacy against Fusarium wilt. The application of Gl006 conidia suspended in water or its own supernatant, as well as the application of Gl006-supernatant, significantly reduced the progress of the disease, in a similar way to the combination of components of the two biocontrol agents (Fig. 8, Table 2). The combination of Bs006-supernatant with Gl006-conidia showed synergy in the reduction of the disease severity progress at 50 days after the application of treatments (Table 2).

**Table 2:** Synergy factor (SF) based on the efficacy of Bs006 and Gl006 against Fusarium wilt incidence (INC) and severity (SEV).

Treatment	AUDPC 50 dai							
	Efficacy INC (%)	Expected efficacy	SF	Interaction	Efficacy SEV (%)	Expected efficacy	SF	Interaction
Bs006 (Sup + Cel)	13,1	70,5	0,18	Antagonistic	0,0	49,4	0	Antagonistic
Bs006 (Sup)	34,4	—	—	—	2,0	—	—	—
Bs006 (Cel)	55,0	—	—	—	48,4	—	—	—
Gl006 (Sup + Con)	70,5	83,1	0,85	Antagonistic	65,0	75,2	0,9	Antagonistic
Gl006 (Sup)	57,4	—	—	—	44,9	—	—	—
Gl006 (Con)	60,3	—	—	—	55,0	—	—	—
Bs006 (Sup + Cel) + Gl006 (Sup + Con)	42,1	74,4	0,56	Antagonistic	53,5	65,0	0,8	Antagonistic
Bs006 (Cel) + Gl006 (Sup)	17,3	80,8	0,21	Antagonistic	18,5	71,6	0,3	Antagonistic
Bs006 (Cel) + Gl006 (Con)	66,1	82,1	0,80	Antagonistic	61,6	76,8	0,8	Antagonistic
Bs006 (Sup) + Gl006 (Con)	67,7	74,0	0,91	Antagonistic	71,5	55,9	1,3	Synergistic
Bs006 (Sup) + Gl006 (Sup)	62,1	72,1	0,86	Antagonistic	11,1	46,0	0,2	Antagonistic

Biological control agents *B. velezensis* Bs006 and *T. virens* Gl006 were applied after transplant at 10^8^ cfu.mL^−1^ and 10^6^ conidia.mL^−1^, respectively. Supernatant of Bs006 and Gl006 were applied at 8.5% and 0.25%, respectively. Con = Conidia, Cel = cells, Bs = *B. velezensis* Bs006, Gl = *T. virens* Gl006. Sup = cells-free supernatant.

## Discussion

In most of the published studies, initial screenings of mixtures of biological control agents were carried out under laboratory conditions, on synthetic media used regularly for the growth of bacteria and fungi, such as nutritive medium, LB or PDA. According to Weller (1988)^19^, in several cases the best biocontrol agents *in planta* are not those showing the highest inhibition of pathogens *in vitro* and that is why new bioassays with conditions close to natural ones have been designed, in which fragments or the entire host are used.

In this work we found that the culture medium influenced the response of the compatibility between *T. virens* Gl006 and *B. velezensis* Bs006, since the synthetic media favored the growth of the bacteria, which completely inhibited the fungus growth. Contrary to what was observed in the medium containing soil solution and root exudates from cape gooseberry, in which only Gl006 grew. In this respect, it is known that *Trichoderma* spp. colonizes nutrient-poor environments, and even nutritional stress may be a prerequisite for the expression of important traits in their biocontrol activity, like some cell-wall degrading enzymes^20^. Unlike to what was found in soil solution medium, Gl006 and Bs006 grew well in the medium containing synthetic root exudates, which contain greater availability of nutrients than the soil solution medium.

In this study, it was found that Bs006-supernatant added to culture medium at 1 and 20%, tend to stimulate the growth of Gl006 during the first 24 h of contact under *in vitro* conditions (Fig. 1, Supplementary material - Figure S1a). In the same way, exposure of Gl006 conidia to Bs006 supernatant for up to 6 hours, did not affect its antagonistic activity or its viability, suggesting high compatibility among Gl006 with the compounds produced by Bs006 and excreted to the medium, indicating the high potential to use these two biocontrol agents in consortium. In fact, the exchange of metabolites and communication signals among members in a consortium could increase or improve the performance of an artificial consortium^21^. It was observed that exposing Bs006 to Gl006-supernatant negatively affected the growth of the bacteria (Fig. 2, Supplementary material - Figure S1b), its ability to form biofilms (Fig. 5) and its biocontrol activity (Fig. 8, Table 2), in a concentration dependent manner, although it did not affect its viability and neither its antagonistic ability. This also suggests that Bs006 can achieve the functions of biocontrol in the rhizosphere in Gl006 presence, considering that the concentration of antimicrobial compounds of Gl006 or *Trichoderma* spp. are much lower in natural conditions than those evaluated *in vitro* in the present work.

The success of biopesticides based on microorganisms depends largely on the ability to actively colonize the target site by the introduced microorganisms^22^. Conidia germination is among the critical features in the process of colonization and survival of a fungus in the rhizosphere, where the production of antibiotics and enzymes, like the cell-wall degrading enzymes could favor its competitive ability^23,24^. It is known also that the formation of biofilms by plant growth promoting rhizobacteria (PGPR) and specifically by *B. velezensis*, is associated with root colonization. PGPR form micro-colonies or biofilms in sites where there is high concentration of root exudates. In these biofilms, bacteria communicate to each other to perform in a coordinated way and has been related to the induction of resistance^25^. In this work we found that the contact of Gl006 conidia with Bs006 did not affect the conidial germination, so this direct interaction would not affect the establishment of the fungus in soil. Likewise, high concentrations of Gl006 supernatant were required to affect to Bs006, which is considered a positive result, since concentration of secreted compounds by *Trichoderma* spp. to the environment in natural conditions, probably is much lower than in the concentration in the supernatant used here which is a dilution (0.25% or 1 × 10^6^ conidia mL^−1^) of the stock inoculum harvested from solid state fermentation (4 × 10^8^ conidia mL^−1^).

Triveni, Prasanna, & Saxena^26^ showed that the mycelium of *Trichoderma viride* can behave like a matrix used by bacteria *P. fluorescens, Azotobacter chrococcum* and *B. subtilis*, to adhere and form biofilms on the fungus surface. The microscopic observations made in the present work allowed to observe this same phenomenon, in which the spores of Bs006 were adhered to conidia of Gl006 after three days contact. It has been described how fungi can contribute to the migration of bacteria as helpers, stimulating their adherence and movement in the soil, as in the case of *Burkholderia terrae* forming biofilms on the hyphae of *Lyophyllum* sp.^27^. This relationship is beneficial for bacteria, considering that movement of bacteria in the soil can be restricted and thus they can reach new microhabitats due to the presence of air among the soil particles^28^. Moreover, it has been found that during biofilm formation by bacteria on fungi structures, bacteria obtain nutrients from exudates secreted by the fungus or released content after inducing lysis of fungus cells or both microorganisms could have a synergistic action in which they need to break complex substrates in the soil^26^. In the present study we found that the interaction between Gl006 and Bs006 was not negative for the fungus, considering that the adherence of the bacteria did not affect Gl006 conidia germination and growth, and physical damage was not observed.

Considering that the concentration of antimicrobial metabolites of *B. velezensis* strains in the rhizosphere is low, its direct activity in the suppression of soil-borne phytopathogens is doubtful^29^. However, in the present work neither the application of Bs006-supernatant nor the suspension of Bs006-cells on its supernatant were effective against Fusarium wilt, despite the application of higher concentrations of antimicrobial metabolites than those that probably exist in the rhizosphere. Suggesting there are other factors in the rhizosphere or in the plant that influence the antagonistic function of these metabolites synthesized by Bs006 or that these metabolites influence in some manner the host response or affect the native microflora in favor of *Fusarium* colonization and infection^2^.

Based on our results, the synergy between Gl006 and Bs006 to control Fusarium wilt could be attributed to the direct action of metabolites contained in Bs006-supernatant on Foph, which could act as a complementary mechanism of action by Gl006. In supernatants and cells interaction experiment it was observed that by eliminating Bs006 cells from the consortium, that is, combining Bs006-supernatant with Gl006-conidia, a high efficiency to control the disease severity was obtained (72%). It has been found that the supernatant of fermentation of Bs006 in LB broth for 48 hours, contained homologous compounds of the cyclic lipopeptides fengycins, iturins and surfactins^2^, and high production of proteases and cellulases, Izquierdo-García and Moreno-Velandia (unpublished). However, these compounds present in the broth are not enough to completely control Fusarium wilt in cape gooseberry considering that in the present work the application of only supernatant treatment reduced the disease incidence by 34% and the severity by 2%. Furthermore, Gl006-conidia reduced Fusarium wilt incidence by 60% and severity by 55%, activity that could be associated mainly with competition for nutrients and space, in addition to the direct action of cell wall degrading enzymes (CWDE) against Foph. On the other hand, Bs006-cells reduced the disease incidence by 55% and the severity by 44%, which allowed to see that, Gl006 cells and Bs006 cells showed high efficacy both individually and as a mixture (66% on both incidence and severity). However, the synergistic effect to control the disease was not attributed to the presence of Bs006-cells in consortium, it could be attributed to direct interaction between Bs006-supernatant with Gl006-conidia, probably due to direct mechanisms of action in a complementary way against Foph.

*Trichoderma* spp. and *Bacillus subtilis/amyloliquefaciens* complex are within the most studied microorganisms in biological control of phytopathogens. The ecological functions of these microorganisms besides biocontrol, such as biodegradation, mineralization, plant growth promotion, stablish the balance in soil for conventional and organic agriculture by increasing the productivity in an agroecosystem^30^. Both microorganisms are highly resistant to adverse environmental conditions, *Trichoderma* spp. have long been known to be a soil-borne fungus, and they are certainly common in the soil^31^, can colonize the surface and inside the plant as endophytes^31,32^, is ubiquitous and highly diverse, *Trichoderma* spp. are the culturable fungi most prevalent in soil studies *in vitro*^32^. Moreover, *Trichoderma* spp. can act as a biofertilizer solubilizing mineral nutrients^33^, has a relatively faster metabolic rates, and secrete anti-microbial secondary metabolites^34^. *Trichoderma* spp. and *Bacillus* spp. also have been studied for their capability in remediation and amelioration of pollutants in the environment^35,36^. *Bacillus velezensis/amyloliquefaciens* complex are highly known as good producers of cyclic lipopeptides (CLPs) that display multiple versatile functions in the interactions with co-existing organisms, including bacteria, fungi, oomycetes, protozoan predators and plants, and contribute greatly to its biological control activity in the colonization process and in the direct control against plant pathogens^37,38^. These characteristics make them ideal candidates to be employed as a consortium in an agroecosystem to control *Fusarium* wilt disease, but they can also offer other services that can be used in the agroecosystems.

About mechanisms of action of Gl006 and Bs006, we have observed that Gl006 synthesizes CWDE such as chitinases (N-acetyl-glucosaminidase and chitobiosidase), proteases, and β,1-3,glucanase, while Bs006 synthesizes cyclic lipopeptides, chitobiosidase, proteases, and β,1-3,glucanase in presence of Foph and individually as well as in consortium. When Gl006 and Bs006 were evaluated in culture media with natural and artificial root exudates, the activity of each one was maintained but we don’t have found an additive or synergistic effect. On the other hand, we evaluated the ability for induce resistance in cape gooseberry under a split root model and found similar results in control of the disease, Izquierdo-García and Moreno-Velandia (unpublished).

The success of a microbial consortium depends on the individual ability of BCA and their mutual interaction^21^. However, it has been found that the biocontrol effect in a consortium can be due to a predominant microorganism in the mixture^39^. In this study, it was found that neither of the two microorganisms negatively affected the other under nutritional conditions similar to those in the rhizosphere (Fig. 7).

Adhesion of *B. velezensis* spores to the surface of *T. virens* conidia without causing damage to the fungus, suggests a compatible interaction between both biocontrol agents. Supernatants from liquid culture of *B. velezensis* could stimulate the biocontrol activity of *T. virens*, while supernatant of *T. virens* reduced *B. velezensis* growth. However, considering that concentrations of metabolites synthesized by these microorganisms in soil could be lower than those synthesized in culture medium, our results suggest that metabolites that are present in supernatant do not affect its biocontrol performance *in vivo*. We found that biocontrol agents *T. virens* Gl006 and *B. velezensis* Bs006 grew in a compatible way on artificial exudates, cape gooseberry root exudates and on the surface of cape gooseberry seeds and roots, suggesting that they could be used in consortium as a biocontrol treatment in cape gooseberry plants.

*T. virens* Gl006 and *B. velezensis* Bs006 are two native soil inhabitants microorganisms and their compatibility as a consortium has been demonstrated in this study, where it is important to maintain the concentrations of both cells and supernatants in favor of their compatibility. Additional studies are required to know molecular interactions of these microorganisms for a better understanding of their compatibility in the rhizosphere.

## Materials and methods

### Microorganisms and culture conditions

*F. oxysporum* f. sp. *physali* strain Map5 (hereafter referred as Foph) and biological control agents (BCA) *Trichoderma virens* (Gl006) and *Bacillus velezensis* (Bs006) were stored at −80 °C in glycerol (30%) and peptone (0,1%) sterile solution at the Microorganisms Collection of Corporación Colombiana de Investigación Agropecuaria - AGROSAVIA. Inoculum of Foph was produced following the procedure described by Moreno^2^. Briefly, this fungus was grown for 7 days on sterile potato-dextrose broth (PDB, Difco^®^) with an initial concentration of 1 × 10^6^ microconidia.mL^−1^, under continuous agitation (125 rpm) at 25 °C. After this, microconidia produced were harvested. The concentration of microconidia suspension required for the experiments was adjusted by Neubauer chamber to 1 × 10^6^ microconidia.mL^−1^, with sterile distilled water (SDW) for *in vitro* tests and with tap water for greenhouse tests. Gl006 was reactivated on potato-dextrose-agar culture medium (PDA) and conidia from the second seven-day-old subculture were harvested by scraping with SDW to obtain the inoculum. Bs006 was reactivated in Luria Bertani Agar culture medium (LBA: Tryptone 10 g, NaCl 10 g, yeast extract 5 g and agar 18 g/L). Pre-inoculum was prepared from the second 24 h-old subculture to start the fermentation in LB broth in an initial concentration of 5 × 10^6^ cfu.mL^−1^. Liquid culture of Bs006 was maintained in continuous agitation in orbital shaker at 125 rpm and 30 °C for 48 h. The required concentration of the bacterial suspensions was adjusted by measuring the optical density in spectrophotometer (BIOTEK®) (OD_600 nm_ = 0.5 ~ 2.49 × 10^8^ cfu.mL^−1^).

### Plant material

Commercial cape gooseberry seeds (Semicol^®)^) were used. Seeds were surface disinfected before sowing by dipping in NaOCl solution (3%) during 20 min and then three washes with sterile distilled water. For greenhouse trials 60-day-old seedlings were used, grown in germination trays of 72 alveoli with autoclaved peat (two continuous cycles of 20 min, 15 PSI and 121 °C). Each seedling was transplanted to 600 g of soil substrate: rice husk (3:1 v/v) in black polypropylene bags of 1000 cm^3^.

### *In vitro* evaluations of the effect of supernatants from liquid cultures of Bs006 and Gl006

The potential effects of secondary metabolites secreted in liquid culture on growth, antagonistic activity, conidia germination for Gl006 and biofilms formation for Bs006 were determined under *in vitro* conditions, after exposure of each microorganism to different concentrations of its partner’s supernatant.

#### Production of supernatants from biocontrol agents

To obtain the supernatant of Gl006, the fungus was grown in Erlenmeyer (300 mL) with 150 mL of sterile PDB (Difco®) for five days (125 rpm, 25 °C) with an initial concentration of 1 × 10^5^ conidia.mL^−1^. After incubation, the fermentation broth was harvested filtering by Whatman No. 1 paper and subsequently by sterile 0.22 μm filters (Millipore^®^) and stored at −20 °C until its use. To obtain the supernatant of Bs006, bacteria was grown in sterile LB broth for 48 hours and then was centrifuged. Supernatant was harvested and filtered with sterile 0.22 μm filters (Millipore^®^). Biomass obtained from Bs006 culture was washed twice to eliminate waste of supernatant and resuspended in SDW, homogenizing by shaking in vortex at maximum speed for two minutes, to be used as inoculum in these experiments. On the other hand, conidia of Gl006 were harvested from a seven-day-old culture on PDA, flooding the medium with sterile distilled water and scraping with a sterile metal rake. The suspension obtained was shaken in a vortex at maximum speed for 30 seconds and then was filtered with sterile muslin (pore size 0,5 mm) to eliminate pieces of mycelium.

#### Effect of supernatants on the growth of biological control agents

To evaluate the effect of Gl006 supernatant on Bs006 and vice versa (Bs006 supernatant on Gl006) the microorganisms were grown in LB or PDB broth, respectively, supplemented with 1, 5, 10, 20 and 30% of supernatant of the other microorganism in 96-well microplates (Greiner^®^). The initial concentrations of Bs006 and Gl006 were adjusted to 5 × 10^6^ cfu.mL^−1^ and 1 × 10^5^ conidia.mL^−1^, respectively. Liquid media supplemented with the supernatants without microbial inoculum were used as blanks, and liquid media supplemented with sterile PDB or LB medium and inoculated with the biocontrol agents were used as controls. Inoculated microplates were incubated for 48 h at 28 °C for Gl006 and 30°C for Bs006 in continuous agitation. The optical density (OD) at wavelength of 630 nm was measured every hour using a microplate reader (BIOTEK®) managed with Gen5® software. Microscope observations were made to evaluate possible effects on the morphology of microorganisms.

#### Effect of B. velezensis Bs006 supernatant on conidia germination of T. virens Gl006

Conidia of Gl006 [5 × 10^6^ conidia.mL^−1^] were exposed during one or six hours to solution of Bs006 supernatant at concentrations mentioned above prepared in sterile solution of Tween 80 (0.1%). After exposition, Gl006 conidia were separated from the solution by centrifugation (14000 rpm, 5 min), then washed twice and resuspended in sterile distilled water. Nine microdrops-10 μL each were inoculated on sterile water-agar in Petri dishes and incubated for 24 h. The proportion of germinated conidia was recorded under 40X magnification in an optical microscope (Olympus^®^ CX41).

#### Effect of Gl006 conidia and supernatant on Bs006 biofilm formation

To determine the effect of *T. virens* conidia and supernatant on Bs006 biofilm formation, we followed the procedure described by Peeters, Nelis, & Coenye (2008)^40^ and O’Toole (2011)^41^. Briefly, the minimum standard medium M63 for biofilm formation supplemented with magnesium sulfate (Merck^®^), glucose (Sigma^®^) and casamino acids (Difco^®^) was used (O’Toole, 2011). This medium was supplemented with Gl006-supernatant at concentrations of 1, 5, 10, 20 and 30%. Moreover, the effect of co-culture of Gl006 in concentrations of 10^5^, 10^6^ and 10^7^ conidia.mL^−1^ with Bs006 cells (24 h-old in LBA) at 1 × 10^8^ cfu.mL^−1^ was evaluated. In both cases, 100 μL of suspension were added per well in a 96-well microplate (Greiner^®^) and incubated for 24 hours at 30 °C. Nine wells were served for each treatment. The fermented broth and the cells in suspension were eliminated after the incubation, and a rinsing was done by adding 100 μL of NaCl solution (0.85%) per well. Then, 100 μL of methanol (99%) were added incubating for 15 minutes, then was eliminated, and the plate was left to dry in laminar flow chamber. Subsequently 200 μL of violet crystal (for gram staining) were added and incubated for 20 minutes, then the colorant was removed and washed with tap water. 150 μL of acetic acid solution (33%) were added to dilute the adhered dye. Absorbance at 590 nm was recorded using a microplate reader (BIOTEK^®^) and the Gen5^®^ software. The experiment was repeated once.

#### Effect of supernatants on the antagonistic activity of BCA on solid media

Conidia of *T. virens* Gl006 and vegetative cells of *B. velezensis* Bs006 exposed for one and six hours to the reciprocal supernatant solutions prepared in Tween 80 (0.1%) sterile solution as described above, were used to carry out a dual confrontation test with Foph in Petri dishes (90 mm) with PDA medium. The inoculated dishes were incubated for seven days at 28 °C in darkness and then the diameter of Foph colony was measured with a caliper (Fischer^®^). The experiment had five dishes per treatment and was performed twice.

### *In vitro* evaluations of co-cultures of biocontrol agents and the influence of the culture medium on the antagonistic activity

The effect of the culture medium and the combination of Bs006 with Gl006 on its antagonistic activity against Foph were measured by dual confrontation tests. The culture media used were LBA, PDA, PD-LBA (50% each), artificial root exudates (ARE)^37^, root exudates from cape gooseberry (CRE), soil solution (SS), ARE and CRE media prepared on SS (SARE and SCRE, respectively).

Root exudates were obtained from 30 days-old cape gooseberry plants. Plants were grown on peat in rooting trays and 30 days after sowing were uprooted from the substrate and washed with tap water. Subsequently, the aerial part of the plant (stem and leaves) and roots were immersed in NaOCl solution (2%) during 0.2 and 5 min, respectively and washed four consecutive times with sterile distilled water. Disinfected plants were placed in sterile plastic tubes 50 mL (Falcon^®^) with roots immersed in 40 mL of sterile soil solution (filtered by 0.2 μm) and were kept at room temperature (18 °C on average) during five days with photoperiod (12 h artificial light − 12 h dark). Rooting solution was harvested and filtered by 0.2 μm filters and stored at −20 °C until use.

Soil solution was obtained by mixing 500 g of substrate (soil:rice husk 3:1 v/v) with 1000 mL of tap water. This solution was agitated on a rotary shaker at 125 rpm during one hour at room temperature and then was filtered with muslin cloth (0.5 mm pore size), then it was passed through 0.8 μm and 0.22 μm sterile filters.

Suspensions of Bs006 vegetative cells (24 h-old on LBA) and Gl006 conidia (7 days-old on PDA) were prepared at 1 × 10^8^ cfu.mL^−1^ and 1 × 10^6^ conidia.mL^−1^, respectively. Two 10 μL-drops were inoculated at opposite points, 1.5 cm from the edge of the Petri dish. Simultaneously, Foph (10 μL, 1 × 10^5^ microconidia.mL^−1^) was inoculated in the center of the plate. Another set of Petri dishes with culture media were inoculated only with one 10 μL-drop of a joint suspension of Bs006, Gl006 and Foph adjusted to concentrations described above. The inoculated plates were incubated for seven days at 28 °C in darkness and then the diameter of the Foph colony was measured with a caliper (Fischer®).

### Microscopy observations

#### Micro-culture of BCA

For microscopic observations of the interacting cells of *T. virens* Gl006 and *B. velezensis* Bs006, a solid support consisting of root exudates of cape gooseberry and sterile agar (18 g.L^−1^) mixture (CREA) was inoculated with 2 μL-drops of cell suspension from Bs006 48 h-old (spores mainly) and Gl006 7 day-old (conidia) on opposite sides on jellified squares-1,5 cm^2^. The jellified support was incubated in a moistened chamber for 72 hours. After this time, observations were made on an optical microscope (Olympus® CX41) and fluorescence microscope (Olympus® IX71).

#### Combined inoculations of Bs006 and Gl006 in planta

Disinfected cape gooseberry seeds were germinated on jellified nutrient medium (GNM)^37^ incubated in the dark for 15 days. Germinated seeds were inoculated at the crown level with 2 μL of mixture or individual inoculum of *T. virens* Gl006 and *B. velezensis* Bs006 [3 × 10^5^ conidia.mL^−1^ and 3 × 10^7^ cfu.mL^−1^, respectively]. Additional inoculations were also carried out on disinfected seeds. Inoculated seeds and seedlings were maintained on GNM at 25 °C for 48 hours. Samples of 1 cm in length were taken from germinated seeds, including the point of inoculation, non-germinated seeds were also observed by scanning electron microscope (FEI^®^, QUANTA FEG 650) with Field Emission technology. The samples were processed by environmental microscopy of plant tissue at 2–4 °C, 400 Pa of pressure and 49.2 to 56% RH.

### Evaluation of the biocontrol activity of Bs006 and Gl006 under greenhouse conditions

The effect of *T. virens* Gl006 with *B. velezensis* Bs006 consortium, including combinations of biomass-biomass, supernatant-biomass, and supernatant-supernatant (Table 2) against Fusarium wilt of cape gooseberry was evaluated under greenhouse conditions. The wet soil was artificially inoculated with microconidia suspension of Foph to obtain a concentration of 1 × 10^4^ cfu.g^−1^ of soil. Then, cape gooseberry seedlings (60 day-old) were transplanted. Gl006 was adjusted to 1 × 10^6^ conidia.mL^−1^ and Bs006 to 1 × 10^8^ cfu.mL^−1^. Biocontrol treatments were applied to the soil immediately after transplant (30 mL.plant^−1^). Plants were kept for 6 weeks under greenhouse with an average temperature of 25 °C.

For this, Bs006 was grown in LB broth for 48 hours at 28 °C and 125 rpm while Gl006 was grown on PDA for seven days at 28 °C. The fermented broth of Bs006 was centrifuged (15000 rpm, 15 min, 4 °C) to separate biomass from supernatant. Supernatant was harvested and filtered by 0.22 μm filters (Sartorius^®^) and biomass of Bs006 was washed twice with sterile distilled water to remove supernatant residues. Gl006 conidia were harvested by scraping the Petri dish and to obtain the supernatant of this fungus, PDA medium in which the fungus grew was liquefied in ULTRA TURRAX^®^ at maximum speed for two minutes, adding 15 mL of sterile distilled water per Petri dish. The suspension obtained was centrifuged under the conditions described before and the obtained supernatant was filtered by 0.22 μm. For the preparation of the treatments, biomass of each biological control agent was resuspended in a supernatant solution of the reciprocal microorganism (biomass of Bs006 in Gl006 supernatant solution and vice versa). Bs006-supernatant and Gl006-supernatant solutions prepared in tap water at 8.3 and 0.25%, respectively were used to dilute the microorganisms according to the volume of the original suspension required to adjust the biomass concentration for each case to 10^8^ cfu.mL^−1^ for Bs006 and 10^6^ conidia.mL^−1^ for Gl006. Combinations of supernatant-supernatant and conidia-bacteria free of supernatant were included as additional treatments. The application of the treatments was performed immediately after transplant.

An experimental design of randomized complete blocks with four replicates was used. The size of the experimental unit was ten plants. Incidence, expressed as the percentage of diseased plants of the total number of plants in the experimental unit and severity of the disease were measured using the scale described by Moreno^2^ (Supplementary material - Table S1, Figure S5) and presented as the disease intensity index, calculated with the equation ${DII}=(\sum {Si}\ast {Ni})\div(5\ast N)$, where *S*i is the level of severity of the symptoms, *N*i is the number of plants in each severity level *S*i, and *N* is the total number of plants in the experimental unit^42^. Disease recording was made every four days from the expression of the first symptoms and the area under the disease progress curve (AUDPC) was calculated. The efficacy of disease reduction was determined based on the AUDPC values with the equation *E=*((*a* − *b*) ÷ *a*) * 100, where *a* represents the value of the disease in the negative control and *b* represents the disease in a treatment^43^.

To determine whether the efficacy on the disease incidence and/or severity showed by the BCA mixtures was the result of a synergistic, antagonistic or additive effect, the expected efficacy (Eesp) in consortium was calculated using the equations of Abbott^43^ *Eesp* = (*a* + *b*) − ((*a* × *b*)/100) where, *a* represents the efficacy by one component of the consortium and *b* represents the efficacy by the other component. Then the synergy factor (SF) was calculated using the equation ${SF}={Eobs}/{Eesp}$ where, *Eobs* corresponds to the efficacy showed by the consortium^44^.

### Statistical analysis

Data were submitted to normality tests according to Shapiro Wilk (*P* > 0.05) and variance homogeneity with Levene test. Significance was determined with ANOVA analysis in a general linear model (GLM). Duncan multiple range test and Tukey test were used to compare media between treatments. Data were analyzed using the statistical software S.A.S. (9.4: SAS Institute, Cary, NC).

## Supplementary information

Supplementary Information.
