# Supplementary material for: Trichoderma virens Gl006 and Bacillus velezensis Bs006: a compatible interaction controlling Fusarium wilt of cape gooseberry
Source: Sci Rep. 2020 Apr 22;10:6857. doi: 10.1038/s41598-020-63689-y (PMC7176702; doi:10.1038/s41598-020-63689-y)
Supplement: Supplementary file 1 — Supplementary Information. [file 41598_2020_63689_MOESM1_ESM.pdf]

Supplementary material

***Trichoderma virens* Gl006 and *Bacillus velezensis* Bs006: a compatible interaction controlling Fusarium wilt of cape gooseberry**

L.F. Izquierdo-García[a], A. González-Almario[b], A.M. Cotes[a], C.A. Moreno- Velandia[a*]

[a] *Corporación Colombiana de Investigación Agropecuaria – AGROSAVIA, Centro de Investigación Tibaitatá, Km 14 vía Bogotá – Mosquera, Colombia*

[b] *Facultad de Ciencias Agrarias, Universidad Nacional de Colombia, Bogotá, Colombia.*

Corresponding author: C.A. Moreno-Velandia; E-mail: cmoreno@agrosavia.co.

Figure S1.

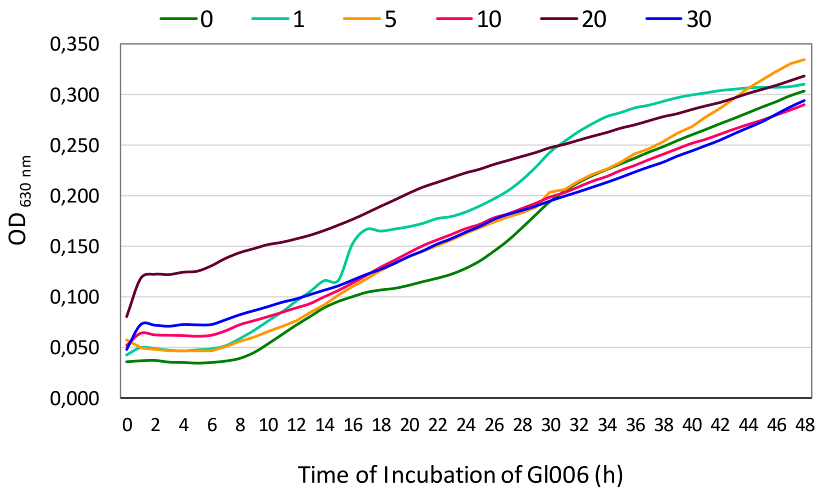

Figure S1a. Growth curves of *T. virens* Gl006 in PDB medium supplemented with cell-free supernatant from *B. velezensis* Bs006 liquid culture. Growth is expressed as optical density ($OD_{630\ nm}$) measured in a microplate reader as a response to concentrations of supernatant solution (1, 5, 10, 20 and 30%). Each point in the curve represents the mean from three samples.

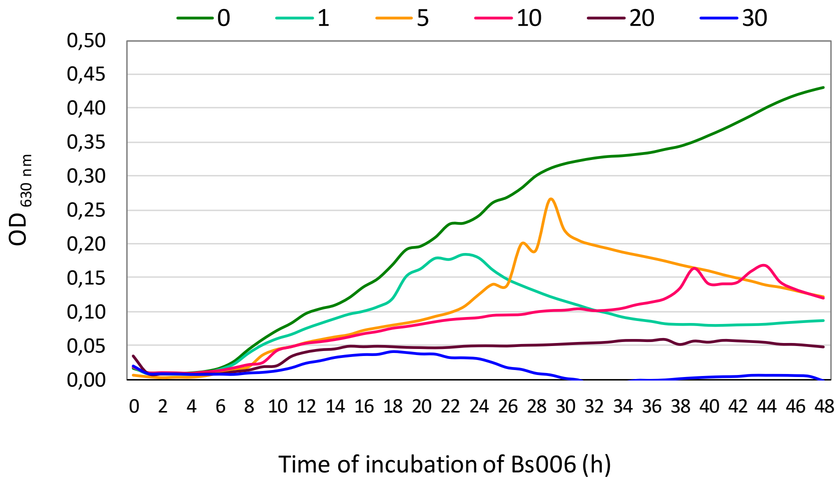

Figure S1b. Growth curves of *B. velezensis* Bs006 in LB medium supplemented with cell-free supernatant from *T. virens* Gl006 liquid culture. Growth of the fungus was expressed as optical density ($OD_{630\ nm}$) measured in a microplate reader as a response supernatant solution (1, 5, 10, 20 and 30%). Each point in the curve represents the mean from three samples.

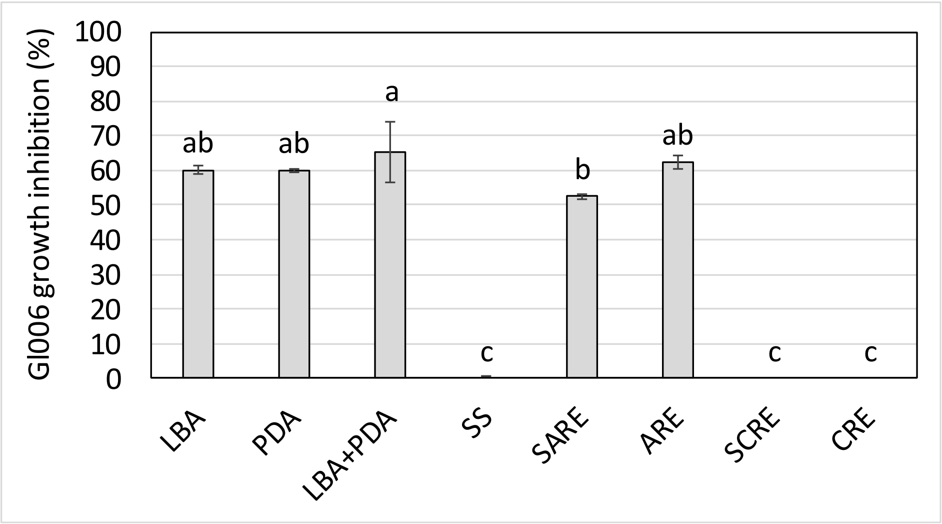

Figure S2. Growth inhibition of *T. virens* Gl006 colony in dual confrontation test with *B. velezensis* Bs006 on different culture media. Bs006 (2 drops 10 µL each at $1x10^8$ cfu mL^{-1}) was inoculated in opposite extremes while Gl006 (one drop of 10 µL at $1x10^6$ conidia mL^{-1}) was inoculated in the center of the Petri dish. Both microorganisms were inoculated simultaneously. Columns with the same letter are not significantly different according to Tukey test (α= 0.05). Bars on the columns represent standard deviation (n=5).

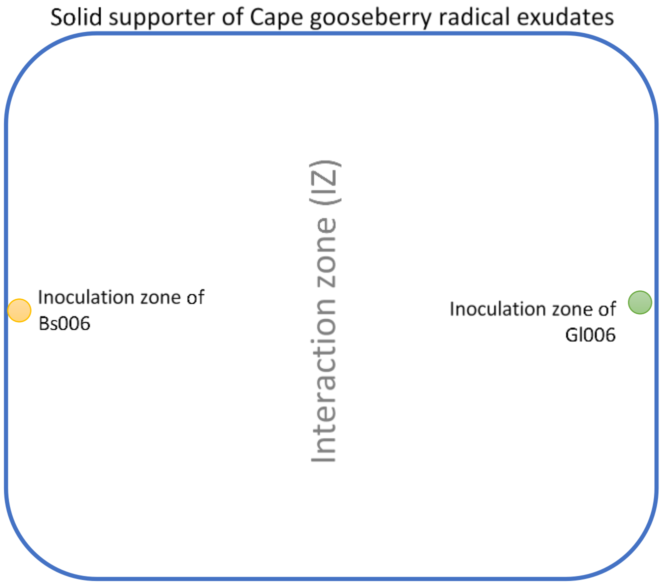

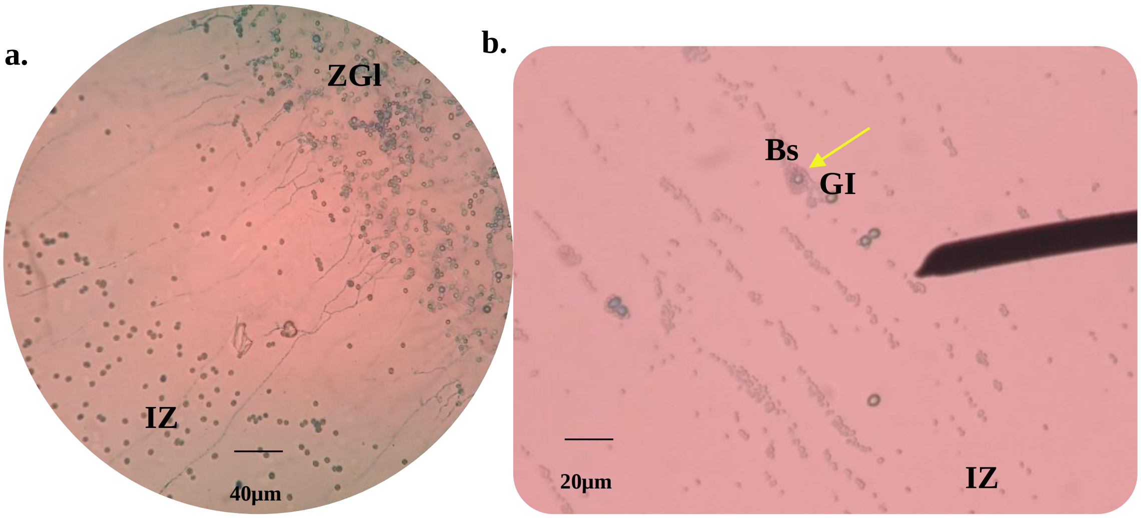

Figure S3. Interaction between *T. virens* Gl006 and *B. velezensis* Bs006 on jellified cape gooseberry root exudates-agar medium. Observations were carried out under optical microscope. **a.** Inoculation zone of *T. virens* Gl006 **b.** Interaction zone (IZ). Yellow arrows indicate biofilm formation of *B. velezensis* Bs006 upon *T. virens* Gl006 conidia after 72 hours contact. **Bs=** Bs006 cells, **Gl=** Gl006 conidia

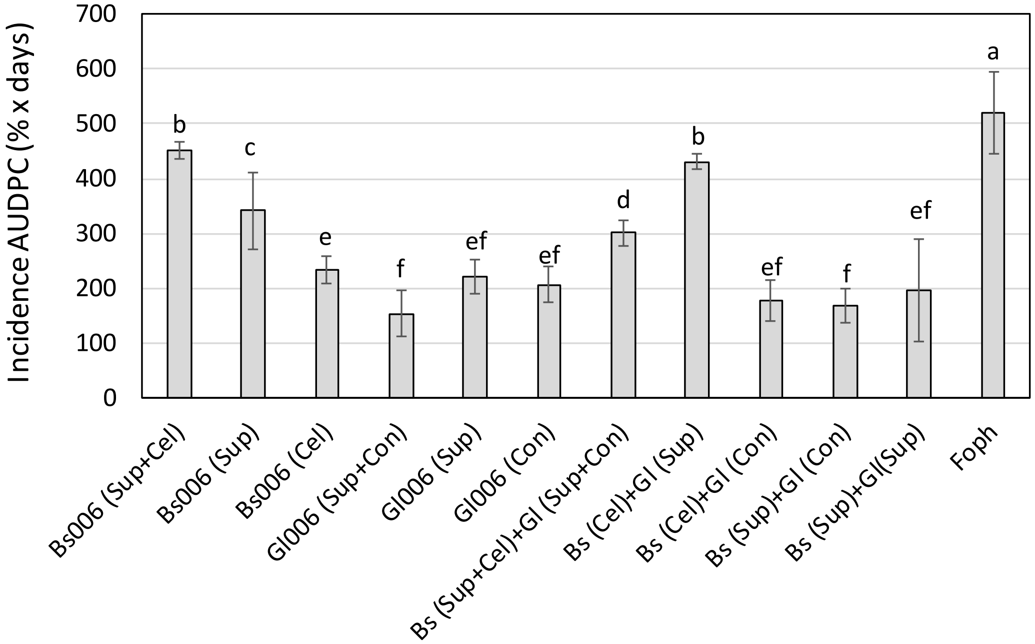

Figure S4. Effect of the combination of cells and supernatants of Gl006 and Bs006 on the progress of Fusarium wilt incidence. Columns with the same letter are not significantly different according to Duncan´s multiple range test (α= 0.05). Con= Conidia, Cel= cells, Bs= *B. velezensis* Bs006, Gl= *T. virens* Gl006. Sup= cell-free supernatant

Table S1. Scale to measure the severity of FW symptoms on cape gooseberry

Level	Symptoms
0	No symptoms of disease, expanded leaves, leaves green and turgid.
1	Mild epinasty, wilt and chlorosis on mature leaves.
2	Epinasty in 30-50% of leaves, moderate wilt and chlorosis on mature leaves and mild in middle layer leaves, obvious growth delay.
3	60-80% leaves with epinasty, obvious loss of turgid, moderate in middle layer, abscission of chlorotic leaves
4	Epinasty in all leaves, severe chlorosis, moderate defoliation, plant in obvious wilt state.
5	Foliar rolling, severe wilt, severe defoliation, stem bending, death plant.

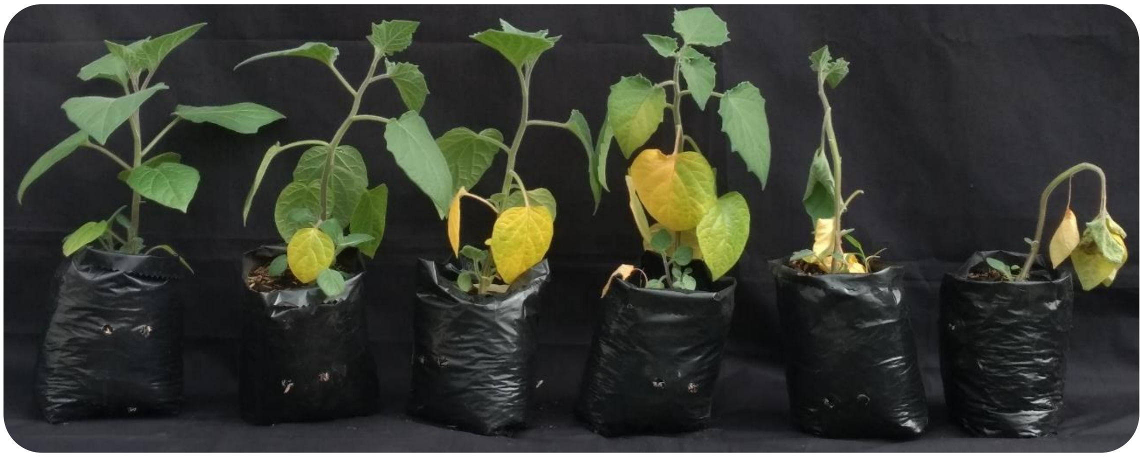

Figure S5. Severity scale of vascular wilt caused by Foph-Map5 on cape gooseberry (Source: author)
